# Use of machine learning-based integration to develop an immune-related signature for improving prognosis in patients with gastric cancer

**DOI:** 10.1038/s41598-023-34291-9

**Published:** 2023-04-29

**Authors:** Jingyuan Ning, Keran Sun, Xiaoqing Fan, Keqi Jia, Lingtong Meng, Xiuli Wang, Hui Li, Ruixiao Ma, Subin Liu, Feng Li, Xiaofeng Wang

**Affiliations:** 1 Department of Immunology, Immunology Department of Hebei Medical University, Shijiazhuang, People’s Republic of China; 2 Department of Pathology, Shijiazhuang People’s Hospital, Shijiazhuang, People’s Republic of China; 3 Department of Laboratory, The Second Hospital of Hebei Medical University, Shijiazhuang, People’s Republic of China; 4 Department of Oncology, Shijiazhuang Fourth Hospital, Shijiazhuang, People’s Republic of China

**Keywords:** Cancer, Computational biology and bioinformatics, Immunology, Biomarkers, Gastroenterology, Oncology

## Abstract

Gastric cancer is one of the most common malignancies. Although some patients benefit from immunotherapy, the majority of patients have unsatisfactory immunotherapy outcomes, and the clinical significance of immune-related genes in gastric cancer remains unknown. We used the single-sample gene set enrichment analysis (ssGSEA) method to evaluate the immune cell content of gastric cancer patients from TCGA and clustered patients based on immune cell scores. The Weighted Correlation Network Analysis (WGCNA) algorithm was used to identify immune subtype-related genes. The patients in TCGA were randomly divided into test 1 and test 2 in a 1:1 ratio, and a machine learning integration process was used to determine the best prognostic signatures in the total cohort. The signatures were then validated in the test 1 and the test 2 cohort. Based on a literature search, we selected 93 previously published prognostic signatures for gastric cancer and compared them with our prognostic signatures. At the single-cell level, the algorithms "Seurat," "SCEVAN", "scissor", and "Cellchat" were used to demonstrate the cell communication disturbance of high-risk cells. WGCNA and univariate Cox regression analysis identified 52 prognosis-related genes, which were subjected to 98 machine-learning integration processes. A prognostic signature consisting of 24 genes was identified using the StepCox[backward] and Enet[alpha = 0.7] machine learning algorithms. This signature demonstrated the best prognostic performance in the overall, test1 and test2 cohort, and outperformed 93 previously published prognostic signatures. Interaction perturbations in cellular communication of high-risk T cells were identified at the single-cell level, which may promote disease progression in patients with gastric cancer. We developed an immune-related prognostic signature with reliable validity and high accuracy for clinical use for predicting the prognosis of patients with gastric cancer.

## Introduction

Gastric cancer (GC) is among the most common malignant tumors. Its incidence in China ranks second among that of all malignant tumors^1^. Worldwide, GC is the fifth leading cause of all cancers and the fourth leading cause of cancer-related mortality^2^. Currently, there are various treatment methods for GC^3^, such as surgery^4,5^, chemotherapy^6^, radiotherapy^7^, targeted therapy^8^, and immunotherapy^9,10^. However, these approaches are not effective in prolonging the life of most patients^11^. Additionally, traditional treatment methods are not effective for patients with advanced stages of GC^12^. At present, anti-programmed cell death 1 (PD-1) monoclonal antibodies, anti-cytotoxic T lymphocyte antigen 4 (CTLA4) monoclonal antibodies, and other immune checkpoint inhibitors (ICIs) are considered innovative treatment strategies for advanced GC^13^. Although studies Keynote-059^14^, Keynote-061^15^, Keynote-062^16^, and attraction-02^17^ have shown a good efficacy of immunotherapy against GC, it seems to be more effective for subgroups with high mutation load, positive Epstein Barr virus, or high microsatellite instability^18^. Many factors, including the tumor immune microenvironment (TME), affect the effectiveness of immunotherapy. There are only a few accurate biomarkers that can predict the response to immunotherapy^19^. Identification of potential prognostic markers and the development of immunotherapy guidelines can aid in designing personalized immunotherapy for patients with GC. Some researchers have suggested that a more in-depth analysis of the complexity of the TME can help reveal efficacious biomarkers that can identify patient populations responsive to immunotherapy^20^. Unfortunately, we still know little about the TME in GC, and we urgently need to identify effective prognostic signatures.

Based on machine learning, prognostic models have been shown to have predictive value in various diseases, including renal cancer^21,22^ colon adenocarcinoma^23^, and endometriosis^24^. Although previous studies have screened immune-related genes of GC to predict the prognosis characteristics, their prediction accuracy is not high^25,26^. In our study, we used a combination of 98 machine learning algorithms to determine the best immune-related prognostic signature for GC and performed external prognostic prediction validation using multiple datasets. Finally, we collected 93 prognostic signatures for comparison. The results showed that our signature was the most effective prognostic biomarker compared to other signatures.

### Methods

### Acquisition and pre-processing of transcriptome data

We downloaded the transcriptome data of 32 normal gastric tissues and 375 GC tissues from The Cancer Genome Atlas (TCGA) website (https://portal.gdc.cancer.gov/). The fragments per kilobase million values were transformed into transcripts per million. Concomitantly, the clinical information corresponding to all patients was downloaded for subsequent analysis.

### Weighted correlation network analysis (WGCNA)

The WGCNA approach was employed to build coexpression networks of genes. To establish a scale-free network, we calculated an optimal soft threshold β. The weighted adjacency matrix was then converted into a topological overlap matrix (TOM), and its corresponding dissimilarity (1-TOM) was computed. The dynamic tree cutting method was utilized to identify modules of coexpressed genes.

### Enrichment analysis

Enrichment analysis of differential genes was performed using the "GSEABase" package, "ClusterProfiler" package and "org.Hs.eg.db" package. The database used for the enrichment analysis was derived from the Gene Ontology (http://geneontology.org/). Use the EnrichGO function for enrichment. If P < 0.05, the pathway was considered to be significantly enriched. "ggplot2" package, "ggpubr" package for visualization.

### Machine learning to build prognostic signatures

In the R(4.2.1) environment, a total of 10 machine learning algorithms, including random survival forest (RSF), elastic network (Enet), Lasso, Ridge, stepwise Cox, CoxBoost, partial least squares regression for Cox (plsRcox), supervised principal components (SuperPC), generalized boosted regression (GBM), and survival support vector machine (survival-SVM) were used. In the process, we used one algorithm to filter the variables and another algorithm to build the prognostic signature. When the final prognostic signature contained less than 5 genes, the signature was considered an invalid signature. A total of 98 combinations of machine learning algorithms were eventually integrated. Finally, Harrell’s concordance index (C-index) was calculated for each signature, and the signature with the highest average C-index value was considered to be the best signature. After calculating the risk score for each patient using the predict function, the optimal cutoff value for the risk score is determined using the surv_cutpoint function in the "srvminer" package. Based on the optimal cutoff value of the risk score, patients are divided into high-risk and low-risk groups.

### Acquisition and pre-processing of single-cell transcriptome data

Single-cell transcriptome data were obtained from the GEO database (GEO registration number: GSE163558; https://www.ncbi.nlm.nih.gov/geo/). Quality control was performed in R(4.1.2) environment using standard single cell processing procedures. The count matrix were read using the Read10X function from the Seurat package (Version 4.0.4), and the latter was further converted to dgCMatrix format. The merge function was used to integrate all individual objects into an aggregate object, and the RenameCells function was used to ensure that all cell labels were unique. We filtered low quality cells with the following filtering criteria: when a gene was expressed in less than 3 cells, the gene was deleted. When the number of genes expressed in a cell was less than 200, the cell was deleted. A global-scaling normalization method (“LogNormalize”) was employed to ensure that the total gene expression in each cell was equal, and the scale factor was set to 10,000. The top 2000 variably expressed genes were returned for downstream analysis using the FindVariableFeatures function. The ScaleData function, “vars.to.regress” option UMI, and percent mitochondrial content were used to regress out unwanted sources of variation. Principal component analysis (PCA) incorporating highly variable features reduced the dimensionality of this dataset, and the first 30 PCs were identified for analysis. Harmony method^27^ was used to remove batch effects between samples. Cells were down-dimensioned using the UMAP method. Clustering analysis was performed based on the edge weights between any two cells, and a shared nearest-neighbor graph was produced using the Louvain algorithm, which was implanted in the FindNeighbors and FindClusters functions. The parameter of resolution in the FindClusters function was tried repeatedly between 0.1 and 1. Cell clustering trees at different resolutions were observed using the clustree function, and the results showed that the clearest clustering results were obtained when the resolution was 0.5. To annotate the cell clusters, differentially expressed markers of the resulting clusters were identified with the FindAllMarkers function using the default nonparametric Wilcoxon rank sum test with Bonferroni correction. All cells were annotated according to cell surface markers and annotated genes used in the relevant literature and CellMarker database^28^ (http://xteam.xbio.top/CellMarker/).

### Identification of high-risk-related phenotypic cells

Scissor algorithm from the "Scissor" package^29^ (2.0.0). By leveraging bulk data and phenotype information, this algorithm automatically selects cell subpopulations from single-cell data that are most responsible for the differences of phenotypes. The novelty of Scissor is that it utilizes phenotype information from bulk data to identify the most highly disease-relevant cell subsets. In our study, high-risk patients and low-risk patients identified in TCGA were treated as two different phenotypes. Based on the transcriptomic data of high- and low-risk phenotypes for all patients, the "Scissor" function was used to associate both phenotypes with each cell in the single-cell data.

### Cellular communication network

Cell–cell interaction analysis was performed based on the “CellChat” (v1.0.0) R package^30^. CellChat has a public repository of ligands, receptors, cofactors and their interactions (http://www.cellchat.org/). The CellChat R package is a versatile and easy-to-use toolkit for inferring, analyzing, and visualizing cell–cell communication from any given scRNA-seq data. The ligand and receptor genes expressed by each cell were projected into a manually selected reference communication network and the probability of communication in each pathway was inferred by gene expression. Finally use the netVisual_bubble function for visualization, with all parameters as default.

### Statistical analysis

All statistical analyses were carried out using R (4.1.2). The statistical methods were all set up according to the corresponding R software.*P* < 0.05 was considered statistically significant. *P < 0.05, **P < 0.01, ***P < 0.001.

### Ethics approval and consent to participate

All methods were carried out in accordance with relevant guidelines and regulations.

## Results

### Cluster analysis of immune subtypes

**Figure 1:**
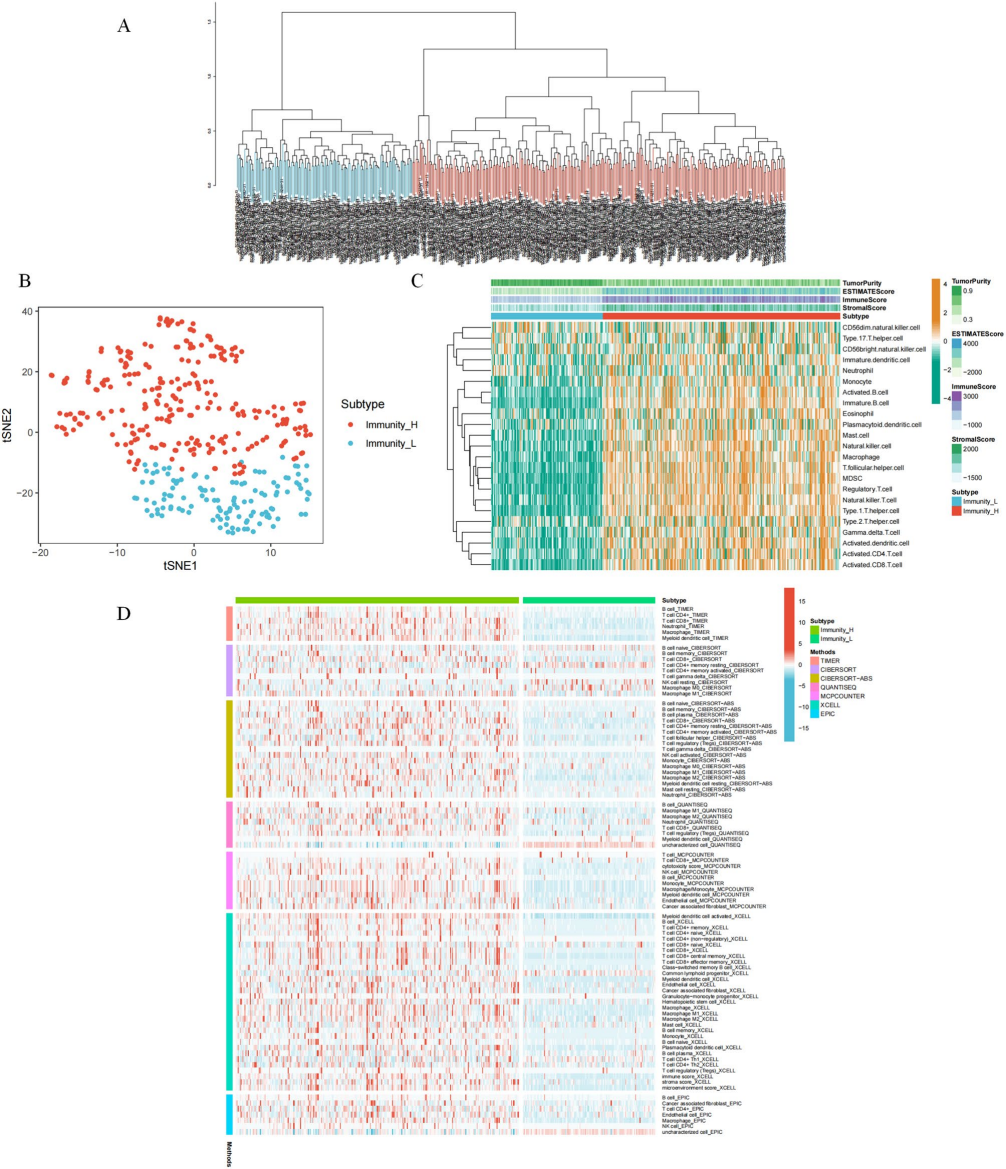
Subtype analysis of patients with TCGA gastric cancer. (**A**) Clustering analysis based on immune cell content. This analysis identified two different patient immunotypes. (**B**) Reduced dimensional analysis of T-SNE based on immune cell content. This figure further validates the immunophenotyping of two different patients. (**C**) Immune cell content calculated by the ssGSEA and ESTIMATE algorithms. (**D**) six algorithms based on TIMER, CIBERSORT, CIBERSORT-ABS, QUANTISEQ, MCPCOUNTER, XCELL, and EPIC for immune cell content assessment. The results show two types of immune patients typed without the bias of the algorithm.

We calculated multiple immune cell scores for each patient with GC using the single sample Gene Set Enrichment Analysis (ssGSEA) method, and based on the scores, we clustered all the patients (Fig. 1A). Furthermore, analysis of all the patients using the T-SNE clustering algorithm showed that the clustering was highly stable in all patients (Fig. 1B). As shown in a heat map, all patients could be divided into two subtypes: one subtype of patients had a higher immune cell score (Immunity_H) and the other subtype had a lower immune cell score (Immunity_L) (Fig. 1C). To demonstrate that our immune cell clustering results are not subject to algorithmic bias, we first used ESTIMATE to verify the plausibility of the ssGSEA results. The results showed that patients in the Immunity_H group had a higher immune score and lower tumor purity compared to patients in the Immunity_L group (Fig. 1C). We then used six algorithms based on TIMER, CIBERSORT, CIBERSORT-ABS, QUANTISEQ, MCPCOUNTER, XCELL, and EPIC for immune cell content assessment. The results all showed that patients in the Immunity_H group had significantly higher immune cell content than patients in the Immunity_L group, which was highly consistent with our clustering results based on the ssGSEA algorithm (Fig. 1D). These findings demonstrate the high stability of our clustering results.

### Identification of immune-related modules

**Figure 2:**
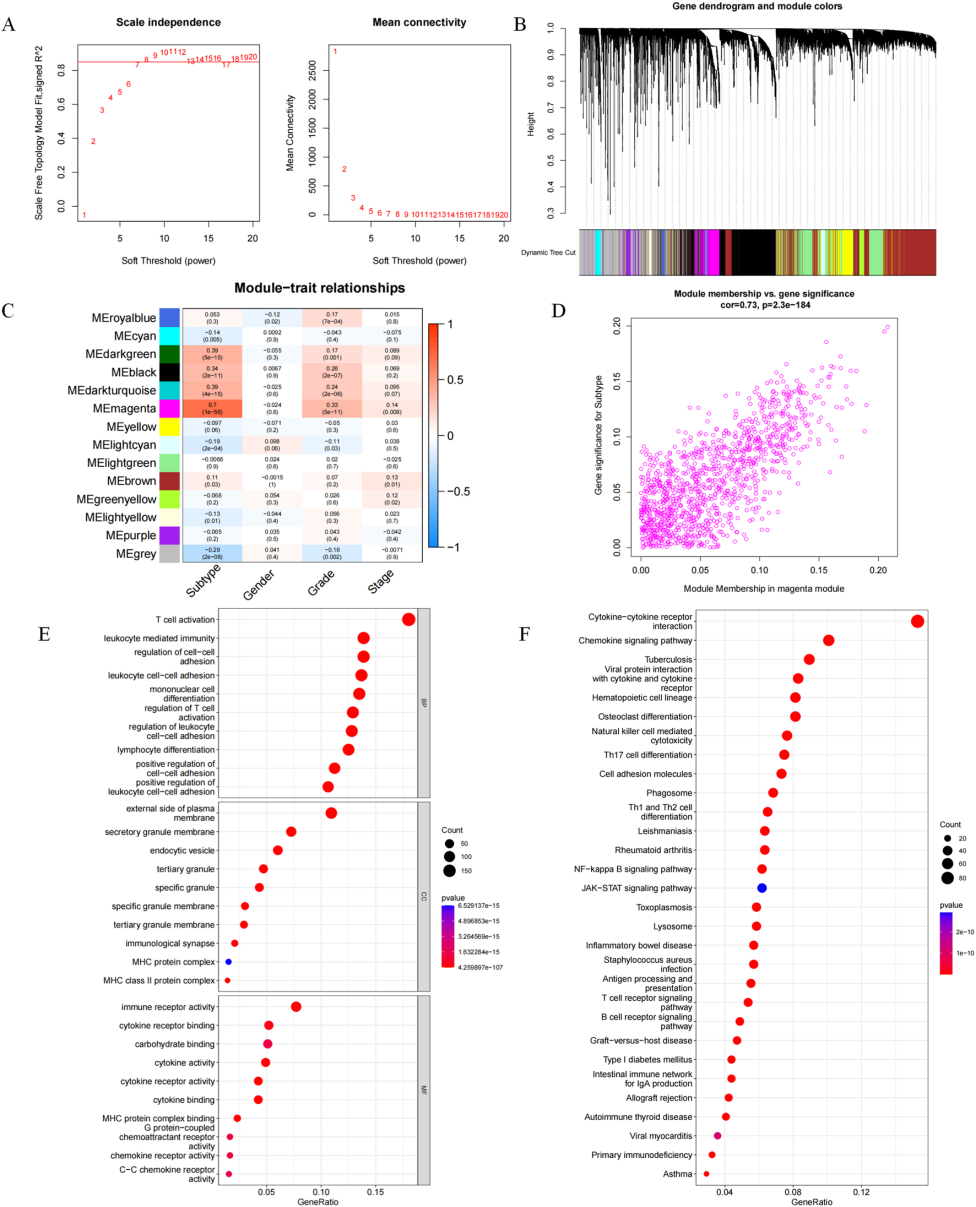
The weighted correlation network analysis. (**A**) Determination of soft thresholds. (**B**) Identification of gene clustering modules. (**C**) Correlation meter analysis between gene modules and phenotypes. Memagenta modules are highly correlated with subtypes (**D**) The correlation coefficient between gene significance (GS) and module membership (MM). (**E**) GO enrichment analysis. (**F**) KEGG enrichment analysis.

In the weighted correlation network analysis (WGCNA), the soft threshold β was set to 8 (Fig. 2A), which provided a suitable power value for the construction of a coexpression network. We identified a total of 14 gene modules, each of which was represented using a different color (Fig. 2B). The correlation between each module and the patient's clinical traits, including sex, grade, stage, and subtype that we clustered based on the ssGSEA method, was evaluated. Among all modules, the correlation between the magenta module and subtype was the highest (Fig. 2C). The correlation coefficient between gene significance (GS) and module membership (MM) reached 0.73 (Fig. 2D), which suggested that the quality of the magenta module construction was superior. Based on these results, we defined the 1104 genes in the magenta module as immune subtype-related genes. To further determine the correlation between these genes and immunity, we performed an enrichment analysis of immune subtype-related genes. The Gene ontology (GO) enrichment analysis results showed that these genes were enriched in T cell activation, leukocyte cell–cell adhesion, regulation of T cell activation, and positive regulation of leukocyte cell–cell adhesion (Fig. 2E). Kyoto Encyclopedia of Genes and Genomes^31^ (KEGG) enrichment analysis results showed that these genes were significantly enriched in cytokine-cytokine receptor interaction, Th1 and Th2 cell differentiation, antigen processing and presentation, B cell receptor signaling pathway, and T cell receptor signaling pathway(Fig. 2F). Together, these results demonstrate a high correlation between immune subtype-related genes and the immune system.

### Generation of signature based on machine learning integration

**Figure 3:**
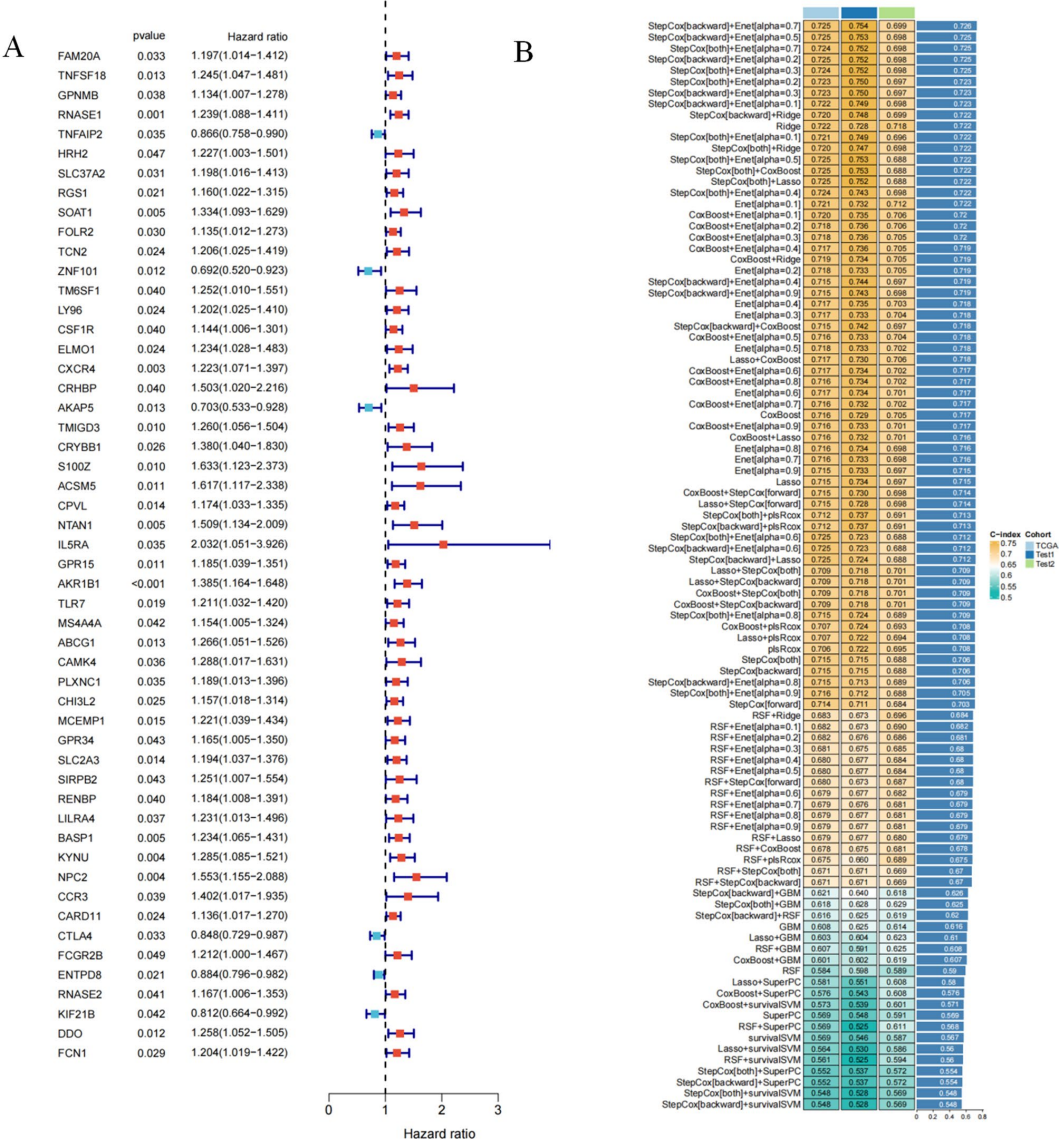
Machine learning integration to build prognostic models. (**A**) Univariate Cox regression identifying 52 prognosis-related genes. (**B**) 98 machine learning integrated prognostic models and their C-index values. Determine StepCox[backward] + Enet[alpha = 0.7] as the best signature.

We performed a univariate Cox regression analysis on the 1104 immune subtype-related genes and identified 52 prognosis-related genes (Fig. 3A), including 6 protective genes (HR < 1) and 46 risk genes (HR > 1). These 52 genes were subjected to our machine learning integration process for establishing immune-related signatures. Specifically, we removed patients with a survival time of fewer than 30 days, after which the remaining 335 TCGA patients with GC were used as the total cohort for subsequent analysis. Meanwhile, to determine the accuracy of our signature, we randomly divided the 335 patients into two cohorts named test 1 and test 2 in a 1:1 ratio. In the total cohort, we fitted 98 prognostic prediction signatures, and the C-index values were calculated in the total, test 1, and test 2 cohorts for each signature. Interestingly, StepCox[backward] + Enet[alpha = 0.7] had the highest mean C-index value (0.726) among all signatures (Fig. 3B). The signature consisted of 24 genes and we named it immune-related signature (IRS). Based on the expression of these 24 genes, we calculated the risk score for all patients. Risk scores = (− 0.2076025 × *TNFAIP2* expression) + (0.1472951 × *SLC37A2* expression) + (0.1714101 × *RGS1* expression) + (− 0.3396804 × *ZNF101* expression) + (− 0.6150795 × *TM6SF1* expression) + (− 0.3724076 × *CRHBP* expression) + (− 0.9322991 × *AKAP5* expression) + (− 0.3562780 × *CRYBB1* expression) + (0.5096191 × *S100Z* expression) + (0.4818817 × *ACSM5* expression) + (0.2350907 × *NTAN1* expression) + (0.5508151 × *IL5RA* expression) + (0.1979289 × *ABCG1* expression) + (0.8638607 × *CAMK4* expression) + (0.2211539 × *MCEMP1* expression) + (0.2176318 × *SLC2A3* expression) + (0.1990519 × *RENBP* expression) + (0.1643554 × *BASP1* expression) + (0.2138879 × *KYNU* expression) + (− 0.2643476 × *CTLA4* expression) + (− 0.2688278 × *FCGR2B* expression) + (− 0.1302975 × *ENTPD8* expression) + (0.2952993 × *DDO* expression) + (− 0.1408996 × *FCN1* expression).

### Accuracy and validity assessment of IRS

**Figure 4:**
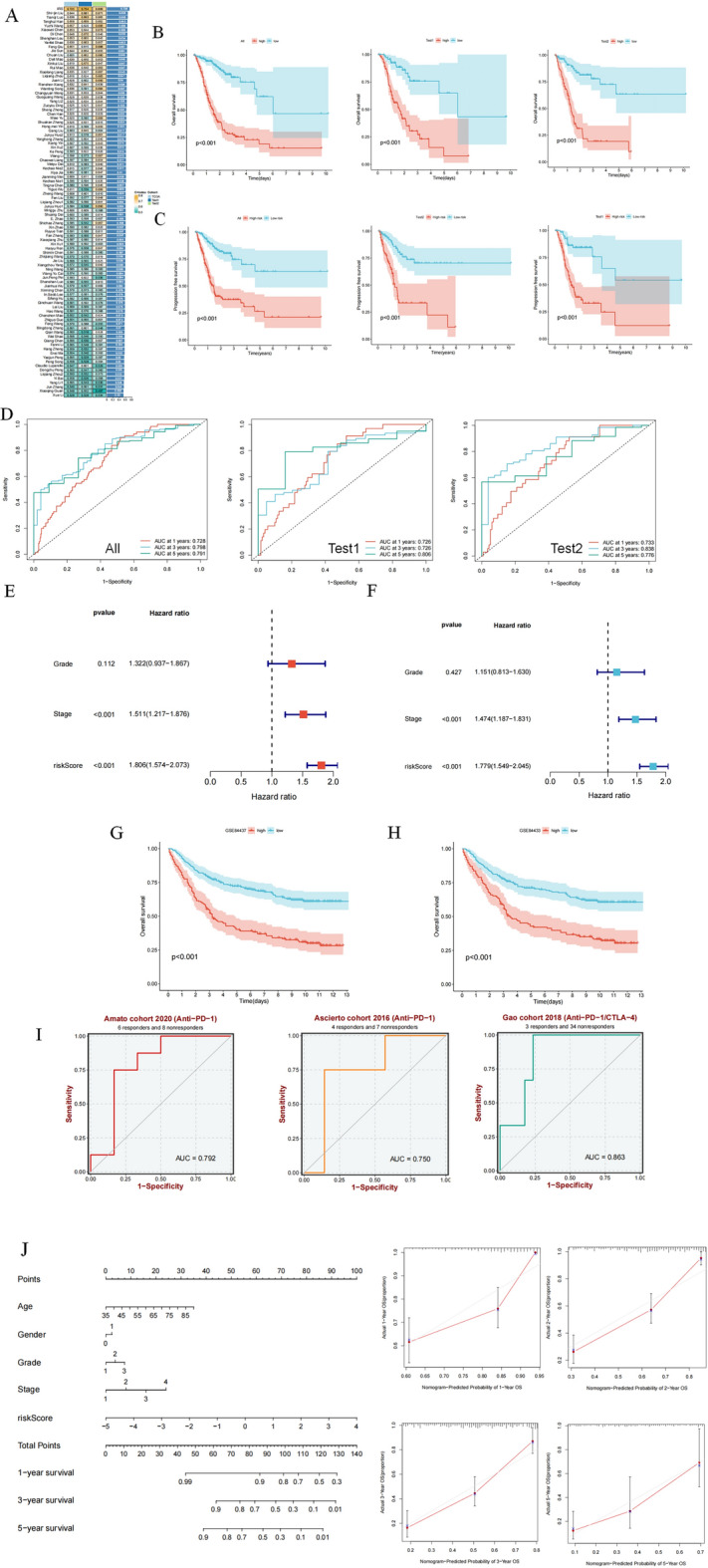
Analysis of the accuracy and validity of IRS. (**A**) C-index values of IRS compared with 93 published prognostic models for gastric cancer. The results demonstrate the outperformance of IRS over published signatures (**B**) OS analysis of IRS in the total cohort, test1 cohort, and test2 cohort. Poorer prognosis for high-risk patients compared to low-risk patients. (**C**) PFS analysis of IRS in the total cohort, test1 cohort, and test2 cohort. Poorer prognosis for high-risk patients compared to low-risk patients. (**D**) 1, 3, and 5 year ROC analysis of IRS in total cohort, test1 cohort, and test2 cohort. (**E**) Univariate Cox regression analysis of IRS and clinical characteristics. (**F**) Multivariate Cox regression analysis of IRS and clinical characteristics. (**G**) OS analysis of IRS in GSE84437. (**H**) OS analysis of IRS in GSE84433. (**I**) ROC analysis of IRS differentiating between responding and non-responding patients in an immunotherapy cohort. IRS has predictive value for patient response to immunotherapy. (**J**) Risk scores combined with clinical characteristics of the nomogram. The calibration curve proves the accuracy of this nomogram.

Notably, a large number of machine learning-based prognostic prediction signatures have been developed in recent years. There is a diversity of these signatures in terms of research perspectives, such as pyroptosis, cuproptosis, ferroptosis, EMT conversion, hypoxia, metabolism, aging, and immune response. To determine the superiority of the IRS, we collected 93 published prognostic signatures and calculated the C-index values for these signatures (Fig. 4A). Importantly, IRS showed the highest C-index values for the total, test 1, and test 2 cohorts compared to these signatures. This suggests that the IRS has robust accuracy. Next, we determined the optimal cutoff value based on the risk score of each patient using the "survminer" package. Kaplan–Meier analysis showed that the overall survival of high-risk patients in the total, test 1, and test 2 cohorts was significantly worse than that of low-risk patients (Fig. 4B). Additionally, we found that progression-free survival (PFS) was significantly worse in high-risk patients in the total, test 1, and test 2 cohorts compared to that in low-risk patients, demonstrating the value of IRS for predicting PFS as well (Fig. 4C).The receiver operating characteristic curve (ROC) analysis showed that the area under the curve (AUC) values for the total cohort were 0.728, 0.798, and 0.791 at 1, 3, and 5 years, respectively. Meanwhile, the AUC values for the test 1 cohort were 0.726, 0.726, and 0.806, and for the test 2 cohort were 0.733, 0.838, and 0.766 at 1, 3, and 5 years, respectively (Fig. 4D). The results of univariate Cox regression (Fig. 4E) and multivariate Cox regression analysis (Fig. 4F) showed that risk score and stage were the two independent factors affecting prognosis, with risk score having the largest HR value. For the purpose of external validation, we introduced the GSE84437 and GSE84433 datasets for external validation of the prognostic effect. The results showed that in both GSE84437 and GSE84433 datasets, high-risk patients had worse overall survival (OS) than low-risk patients (Fig. 4G,H). Finally, we assessed the value of IRS in immunotherapy. AUC values were higher than 0.75 in two anti-PD-1 treatments and one anti-PD-1/anti-CTLA-4 (Fig. 4I), suggesting that IRS is relevant for predicting sensitivity to immunotherapy in patients with gastric cancer. In conclusion, our results suggest that IRS has excellent stability and validity. To improve the clinical value of this study, we created a nomogram based on risk scores and clinical characteristics to facilitate clinical translation (Fig. 4J). The calibration curve showed that the nomogram had good accuracy at 1, 2, 3, and 5 years.

### IRS combined with single-cell analysis identifies communication perturbations in high-risk cells

**Figure 5:**
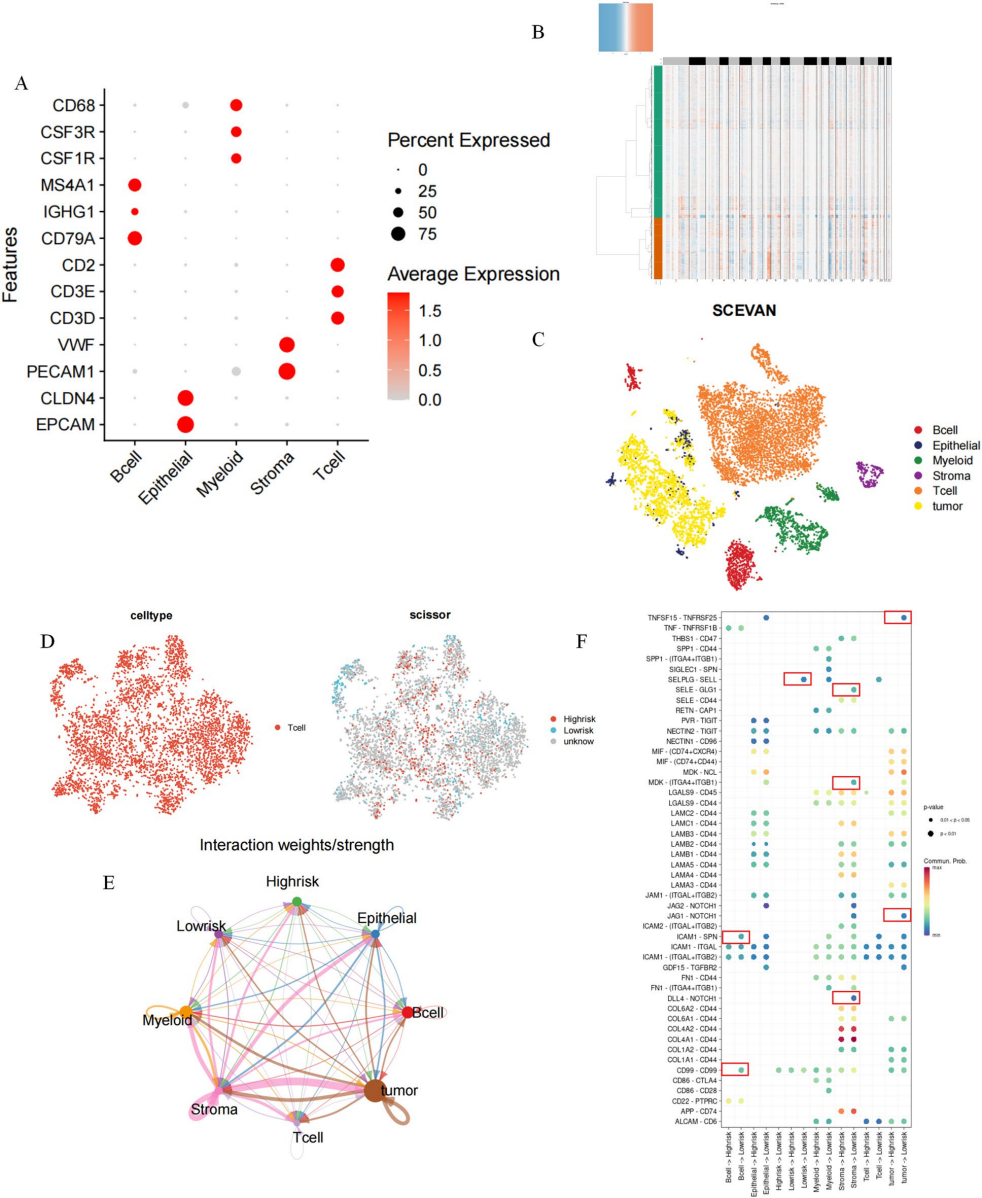
Single-cell analysis of IRS. (**A**) Expression of cellular annotated genes. (**B**) Copy number changes in tumor cells and normal cells. (**C**) Cell types after annotation of all cells. (**D**) Identification of high-risk and low-risk T cells. (**E**) Cellular communication analysis of the landscape. (**F**) Analysis of ligand-receptor interactions between different cell types.

Cell-to-cell communication plays a crucial role in understanding the complexity of the tumor immune microenvironment. For instance, in cancer, tumor cells interact with various immune cells and stromal cells in the tumor microenvironment. These interactions can shape disease progression and response to therapy. However, this is not captured by bulk transcriptomic data. Luckily, single-cell sequencing technology enables the possibility of uncovering intricate and complex interactions between cells. Single-cell analysis of tumors and immune cells can provide an in-depth understanding of the molecular mechanisms of tumor-immune cell interactions, which can offer information for the development of new immunotherapies. Hence, we acquired and processed single-cell sequencing data from the tumor sites of three patients with GC.Here, a total of 10,234 cells passed quality control. All cells were annotated according to cell surface markers (Fig. 5A). Five cell types were present, namely B cells, epithelial cells, myeloid cells, stroma cells, and T cells. For epithelial cells, we identified 2634 malignant cells using the "SCEVAN" package^32^, which has been shown to be significantly more accurate than “inferCNV” and “copyKAT”. Figure 5B shows the landscape of copy number variation in normal and tumour cells. Cell down-dimensioning and visualization were performed using T-SNE (Fig. 5C). Among the immune cells, T cells were the most numerous. To identify the T cells that contribute to the high-risk disease phenotype, the “scissor” package was used to correlate bulk sequencing data with single-cell sequencing data. This method uses single-cell data and phenotypic information to identify subpopulations of cells. Using bulk sequencing data and its annotated information with various phenotypes, the algorithm automatically selects cells that are highly correlated with the phenotype. We considered high-risk and low-risk in patients as two phenotypes, associating both phenotypes with T cells that contribute to the high-risk disease phenotype. We successfully identified a total of 507 high-risk cells and 365 low-risk cells (Fig. 5D). We then used the "cellchat" package to analyze the differences in cellular communication networks between the high-risk and low-risk cells (Fig. 5E). Multiple signaling perturbations were found between high-risk T cells, low-risk T cells, tumor cells, and normal epithelial cells (Fig. 5F). JAG1-NOTCH1 and TNFSF15-TNFRSF25 signals were present between tumor cells and low-risk T cells, and absent between tumor cells and high-risk T cells. The autocrine SELPLG-SELL signaling present in low-risk T cells was lost in high-risk T cells. Furthermore, we found some alterations between immune cells. For example, disappearance of DLL4-NOTCH1, MDK-(ITGA4 + ITGB1), and SELE-GLG1 signaling between stromal cells and high-risk T cells.Disappearance of ICAM1-SPN signaling with CD99-CD99 signaling in B cells and high-risk T cells.

## Discussion

With the application of immunotherapy to GC, the treatment of GC has entered a new era. However, not all patients with GC can benefit from immunotherapy. Several studies have attempted to identify better immune-related characteristic genes that affect the prognosis of patients but have not been very successful^25,26^. As early as last century, it was proposed that the immune microenvironment of gastric cancer is the key factor affecting the prognosis of gastric cancer patients^33^. The level of infiltration of T cells, macrophages and various immune cells affects the prognosis of patients with gastric cancer^34,35^. Since 2019, many studies have been devoted to the establishment of immune related signature for gastric cancer. These signature not only affect the prognosis of gastric cancer patients, but also affect the efficacy of chemotherapy, immunotherapy and other treatments for patients with gastric cancer^36–38^. Therefore, the current study aimed to find the optimal immune-related prognostic signature for patients with GC.

WCGNA analysis was used to identify 14 gene modules. The evaluation of the correlation between each module and the patient's clinical characteristics showed that among all modules, the magenta module had the highest correlation with the subtype. Therefore, 1104 genes in the magenta module were defined as immune subtype-related genes. Subsequently, these 1104 immune subtype-related genes were used for univariate Cox regression analysis, and 52 prognosis-related genes were identified to establish immune-related signatures. In the total cohort, we fitted 98 prognosis prediction signatures. For each signature, we calculated the C-index value in the total, test 1, and test 2 cohorts, and the signature with the highest average C-index value was considered the best signature. Among all the signatures, StepCox [backward] + Enet [alpha = 0.7] showed the highest average C-index value (0.726). This signature, named in this study as IRS, is composed of 24 genes: *TNFAIP2*^39,40^, *SLC37A2*, *RGS1*^41^, *ZNF101*, *TM6SF1*, *CRHBP*, *AKAP5*^42^, *CRYBB1*, *S100Z*^43^, *ACSM5*, *NTAN1*^44^, *IL5RA*, *ABCG1*, *CAMK4*, *MCEMP1*^45–47^, *SLC2A3*^48–50^, *RENBP*, *BASP1*^51,52^, *KYNU*^53,54^, *CTLA4*^55^, *FCGR2B*^56^, *ENTPD8*, *DDO*, *FCN1*^57^. All of these genes have been mentioned in previous studies to affect the prognosis of patients with GC. Especially, *CTLA4* has been used in the clinic as a target for mature tumor-targeted therapy^58^, which indicates the accuracy of our signature.

A large number of prognosis prediction signatures based on machine learning have been reported in recent literature. In terms of research, these signals have diversity, such as pyroptosis, cuproptosis, ferroptosis, EMT conversion, hypoxia, metabolism, aging, and immune response^36,37,59–64^. We collected 93 published prognostic signatures and calculated C-index values for these signatures. Compared with these signatures, the IRS showed a higher C-index value in the total, test 1, and test 2 cohorts, indicating its high accuracy. Higher TMB has been demonstrated to be associated with better prognosis in patients with GC, which is consistent with our findings^65^. Immune interaction is the key feature of tumorigenesis and the therapeutic target of GC. Stromal cells and immune cells are the main components of TME, and immune and matrix scores are related to the clinical features and prognosis of GC^66,67^. Our results also confirmed that tumor immunity is the most important factor affecting the prognosis of GC patients.

Subsequently, we processed the tumor site single-cell sequencing data of patients with GC and identified 2634 malignant tumor cells. Upon cell dimension reduction and visualization, we noticed that the number of T cells is the largest among immune cells. Recent studies emphasize that several types of tumor infiltrating lymphocytes (TIL) are associated with better disease outcomes for various human cancers^68,69^, It indicates that more CD3+, CD8+ or CD45RO + T cells in tumor tissue are significantly associated with lower frequency of lymph node metastasis, disease recurrence or longer survival of patients. However, tumors have developed many different strategies to escape immune surveillance, such as loss of tumor antigen expression, expression of Fas ligand (Fas-L) or *CD200* that can induce apoptosis of activated T cells, and immunosuppressive cytokine secretion, such as IL-10 or TGF-β, Or production of regulatory T cells, and downregulation or loss of MHC^70^. The change of HLA class I expression occurs in gastric cancer^71^, and may play a role in the clinical process of disease by making tumor cells escape T cell mediated immune response^72^. This intercellular communication may be the main reason for the different prognoses of different patients with GC.

In our study, we used a combination of various machine learning algorithms to construct immune-related prognostic signatures for gastric cancer, and validated the stability and effectiveness of the immune-related signature (IRS) using multiple datasets. We compared IRS with 93 previously published prognostic signatures, and demonstrated that IRS was the most effective prognostic signature. Through the evaluation of IRS, doctors can better understand the patient's prognosis and consider it in their treatment plan, helping to develop more personalized treatment plans and maximize the patient's survival rate. Additionally, we further discovered the predictive value of IRS for the response to immune checkpoint therapy, which is based on the patient's immune gene expression profile and can predict the patient's response to immunotherapy. In clinical practice, the application of IRS can providing more accurate and personalized guidance for patient treatment and management. Importantly, we further revealed cellular communication between high-risk and low-risk T cells at the single-cell level, which provides important reference value for the study of tumor immune microenvironment in gastric cancer patients. However, our study still has some shortcomings. IRS needs to be re-validated in real-world cohorts, and the combination of IRS with clinical features that affect patient prognosis may further improve accuracy. In summary, IRS is a promising tool for clinical prognosis prediction and immune therapy decision-making for GC patients.

## Conclusions

We developed an immune-related prognostic signature with reliable validity and high accuracy for clinical use for predicting the prognosis of patients with gastric cancer.

## Data Availability

Single-cell transcriptome data were obtained from the GEO database (GEO registration number: GSE163558; https://www.ncbi.nlm.nih.gov/geo/). Bulk transcriptome sequencing data were obtained from the TCGA database (https://portal.gdc.cancer.gov/) and GEO database (GSE84437, GSE84433).

## Abbreviations

WGCNA: Weighted correlation network analysis
GC: Gastric cancer
PD-1: Programmed cell death 1
CTLA4: Cytotoxic T lymphocyte antigen 4
ICIs: Immune checkpoint inhibitors
TME: Tumor immune microenvironment
TCGA: The Cancer Genome Atlas
GEO: Gene expression omnibus
C-index: Concordance index
ssGSEA: Single sample gene set enrichment analysis
GS: Gene significance
MM: Module membership
IRS: Immune-related signature
GO: Gene ontology
ROC: Receiver operating characteristic
AUC: Area under the curve
RSF: Random survival forest
Enet: Elastic network
plsRcox: Partial least squares regression for Cox
SuperPC: Supervised principal components
GBM: Generalised boosted regression
survival-SVM: Survival support vector machine
C-index: Concordance index
OS: Overall survival
PFS: Progression-free survival
